# Supplementary material for: Very severe anemia and one year mortality outcome after hospitalization in Tanzanian children: A prospective cohort study
Source: PLoS One. 2019 Jun 20;14(6):e0214563. doi: 10.1371/journal.pone.0214563 (PMC6586275; doi:10.1371/journal.pone.0214563)
Supplement: S1 Table — (DOCX) [file pone.0214563.s001.docx]

S1 Table. Predictors of in-hospital and post-hospital mortality of children with very severe anemia by univariate analysis.

|  | **In-Hospital mortality** | | **Post-Hospital mortality** | |
| --- | --- | --- | --- | --- |
|  | **HR [95% CI]** | ***p*-value** | **HR [95% CI]** | ***p*-value** |
| Demographic Characteristics | | | | |
| Female | 2.06 [0.63 – 6.77] | 0.230 | 0.57 [0.18 – 1.77] | 0.335 |
| Age (months) | 1.01 [0.99 – 1.03] | 0.106 | 1.01 [0.99 – 1.02] | 0.165 |
| Categorical Age | | | | |
| Under 5 years | Reference |  | Reference |  |
| 5-12 years | 4.09 [1.08 – 15.45] | **0.037** | 2.23 [0.83 – 6.00] | 0.111 |
| Lake/pond as water source | 0.44 [0.11 – 1.69] | 0.236 | 0.72 [0.26 – 1.99] | 0.530 |
| Pit latrine at home | 0.95 [0.27 – 3.25] | 0.940 | 1.00 [0.36 – 2.77] | 0.990 |
| Reported on Hospitalization | | | | |
| Fever | 0.47 [0.12 – 1.77] | 0.266 | 1.17 [0.26 – 5.19] | 0.827 |
| Vomiting | 0.35 [0.04 – 2.80] | 0.328 | 2.17 [0.78 – 5.98] | 0.133 |
| Diarrhea | 0.78 [0.16 – 3.64] | 0.759 | 0.19 [0.02 – 1.45] | 0.111 |
| Decreased urine output | 14.92 [4.50 – 49.46] | **<0.001** | 10.36 [1.27– 84.25] | **0.029** |
| Taking herbal medication | 2.07 [0.63 – 6.80] | 0.228 | 0.84 [0.29 – 2.44] | 0.761 |
| Signs on Physical Examination | | | | |
| Temperature (Celsius) | 1.08 [0.61 – 1.91] | 0.785 | 0.56 [0.31 – 0.96] | **0.037** |
| Heart rate (beats per minute) | 1.00 [0.97 – 1.02] | 0.892 | 0.99 [0.96 – 1.01] | 0.422 |
| Systolic blood pressure (mmHg) | 0.95 [0.92 – 1.00] | 0.054 | 1.00 [0.96 – 1.03] | 0.909 |
| Diastolic blood pressure (mm Hg) | 0.89 [0.83 – 0.96] | **0.001** | 0.95 [0.90 – 1.01] | 0.115 |
| Respiratory rate (breaths per minute) | 1.02 [0.98 – 1.06] | 0.180 | 1.02 [0.99 – 1.06] | 0.071 |
| Oxygen saturation (%) | 0.92 [0.86 – 0.99] | **0.025** | 0.88 [0.79 – 0.98] | **0.029** |
| Nutritional Status |  |  |  |  |
| Severe malnutrition^a^ | 1.50 [0.36 – 6.32] | 0.573 | 1.44 [0.36 – 5.76] | 0.605 |
| Moderate malnutrition^b^ | 0.48 [0.05 – 4.13] | 0.507 | 1.11 [0.27 – 4.45] | 0.880 |
| Mild malnutrition^c^ | 0.66 [0.13 – 3.51] | 0.648 | 1.17 [0.33 – 4.16] | 0.803 |
| Glasgow Coma Score | 0.48 [0.26 – 0.90] | **0.022** | 0.76 [0.32 – 1.79] | 0.538 |
| Pallor | ^d^ | **0.031** | 0.84 [0.30 – 2.32] | 0.746 |
| Edema | 6.23 [1.88 – 20.58] | **0.003** | 2.59 [0.83 – 8.05] | 0.099 |
| Laboratory Investigation | | | | |
| Random blood glucose (g/dL) | 0.79 [0.51 – 1.24] | 0.320 | 1.09 [0.88 – 1.34] | 0.385 |
| Estimated glomerular filtration rate (mL/min/1.73m^2^) | 0.99 [0.98 – 1.00] | 0.155 | 1.00 [0.99 – 1.01] | 0.424 |
| Proteinuria by urinalysis | 2.15 [0.63 – 7.35] | 0.222 | 2.21 [0.76 – 6.33] | 0.142 |
| Hematuria by urinalysis | 2.76 [0.59 – 12.80] | 0.193 | 1.79 [0.40 – 7.89] | 0.441 |
| HIV positive | 4.77 [1.02 – 22.27] | **0.046** | 2.15 [0.28 – 16.30] | 0.459 |
| Hemoglobin level (g/dL) | 0.61 [0.31 – 1.19] | 0.152 | 0.70 [0.40 – 1.21] | 0.202 |
| Diagnostic Category | | | | |
| Malaria | NA | NA | 0.67 [0.15 – 2.99] | 0.609 |
| Sickle cell disease | 0.55 [0.11 – 2.56] | 0.450 | 1.47 [0.53 – 4.06] | 0.451 |
| Severe malnutrition | NA | NA | 1.04 [0.13 – 7.89] | 0.969 |
| Diarrheal diseases | 4.30 [0.55 – 33.72] | 0.164 | NA | NA |
| Respiratory infections | 4.50 [0.56 – 35.89] | 0.155 | NA | NA |
| Heart disease | NA | NA | 3.60 [0.47 – 27.41] | 0.215 |
| Cancer | 4.67 [1.00 – 21.64] | **0.049** | 2.24 [0.29 – 16.99] | 0.435 |
| Septic shock | 29.33 [4.90–175.54] | **<0.001** | NA | NA |
| Urinary tract infection | NA | NA | NA | NA |
| Neurologic diseases | NA | NA | NA | NA |

Abbreviations: NA, not applicable

^a^ Weight-for-Height Z score < -3 SD

^b^ Weight-for-Height Z score < -2 and ≥ -3 SD

^c^ Weight-for-Height Z score < -1 and ≥ -2 SD

^d^ Hazard ratio could not be calculated because of perfect predictor. All very severely anemic children who died

in-hospital were noted to have pallor. *P*-value determined from Fisher exact.
